# Supplementary material for: Graphene Oxide-Chitosan Aerogels: Synthesis, Characterization, and Use as Adsorbent Material for Water Contaminants
Source: Gels. 2021 Sep 24;7(4):149. doi: 10.3390/gels7040149 (PMC8544572; doi:10.3390/gels7040149)
Supplement: Supplementary file 1 [file gels-07-00149-s001.zip › gels-1327737-supplementary.pdf]

gels

Article

Graphene Oxide-chitosan Aerogels: Synthesis, Characterization and Use as Adsorbent Material for Water Contaminants

Filippo Pinelli, Tommaso Nespoli and Filippo Rossi *

Department of Chemistry, Materials and Chemical Engineering “Giulio Natta”, Politecnico di Milano, via Mancinelli 7, 20131, Milan, Italy; filippo.pinelli@polimi.it (F.P.); tommaso.nespoli@mail.polimi.it (T.N.)
* Correspondence: filippo.rossi@polimi.it; Tel.: (+39) 02 23993145, Fax: (+39) 02 23993180;

Supplementary Information (SI)

XRD patterns

In Figure S1 we reported the diffraction pattern of the composite graphene oxide-chitosan aerogels. As widely reported in literature, the diffraction pattern of pure graphene oxide has a peak at 2ϑ = 10° corresponding to lattice planes [1]. However, here there was no sharp peak at 2ϑ = 10° for our material indicating the formation of a good composite of graphene oxide in the interior of the chitosan. Moreover the profile that can be observed in the pattern matches with works reported in literature [1,2].

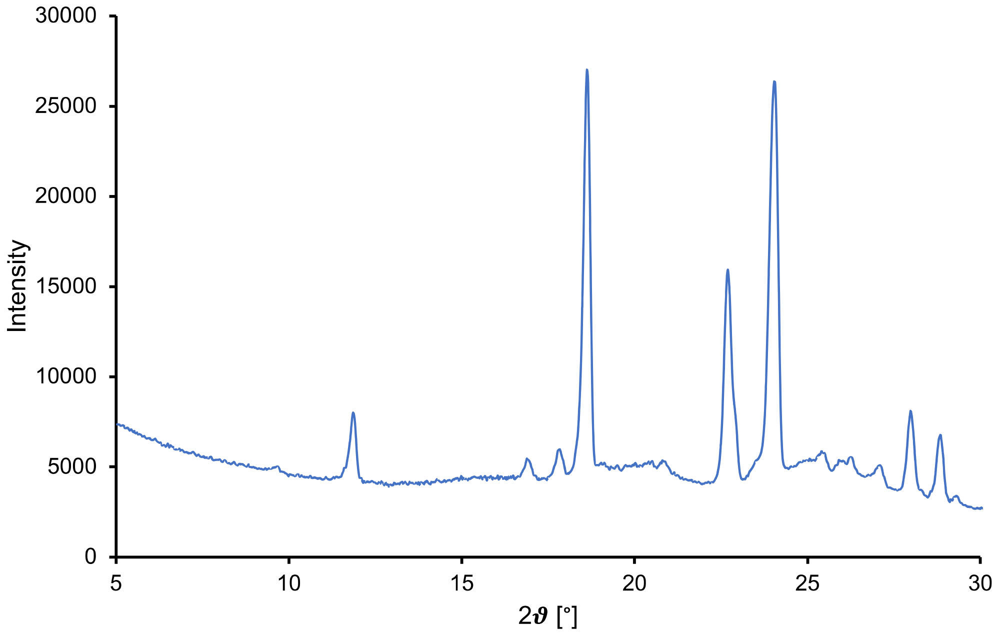

Figure S1. XRD patterns of graphene oxide-chitosan aerogels.

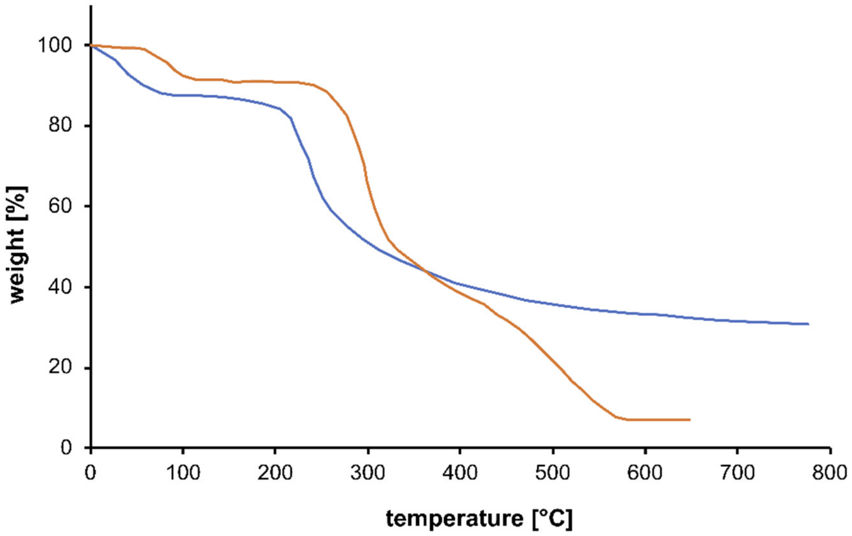

Figure S2. Thermogravimetric analysis of graphene oxide-chitosan aerogels (blue line) and the same device obtained without graphene oxide (red line).

Calibration lines for the employed dyes

In order to evaluate the sorption capacity of the synthetized aerogels, the absorbance of the water was evaluated at each time interval of adsorption to calculate the amount of dye sorbed by the materials and the quantities still present in solution. The quantitative correlation between absorbance data and dye concentration was realized using a calibration line built for each dye. Moreover, to identify the characteristic peaks of each dye we referred to spectrum analysis that can be found in literature [3].

The calibration lines were obtained with the following procedure. Starting from solutions of 90 mg/L, five different solutions with known concentrations were obtained for the dyes thorugh dilutions of the intial one. Measurement at fixed wavelengt were performed and the absorbance value data were plotted against concentration to find the interval of applicability of the Lamber-Beer law and the correlation. Calibration lines Absorbance vs Concentration (mg/L) for the considered dyes are reported iin Figure S3 and S4.

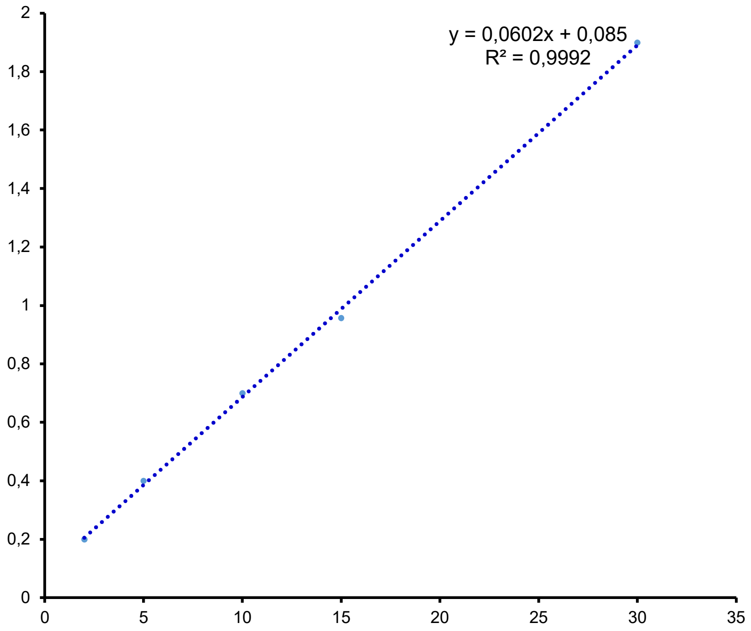

Figure S3. Calibration line for Indigo Carmine.

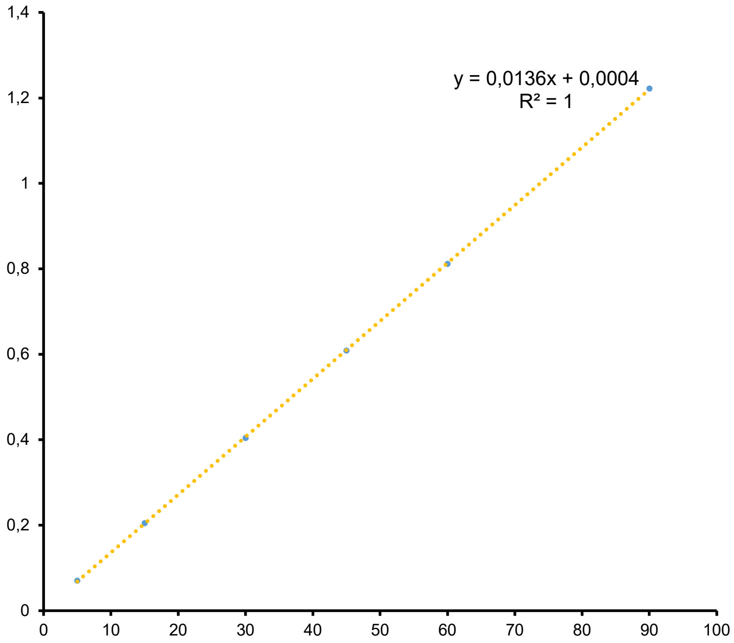

Figure S4. Calibration line for Cibacron Brilliant Yellow.

References

1. Gong, Y.; Yu, Y.; Kang, H.; Chen, X.; Liu, H.; Zhang, Y.; Sun, Y.; Song, H. Synthesis and characterization of graphene oxide/chitosan composite aerogels with high mechanical performance. *Polymers (Basel).* **2019**, *11*, doi:10.3390/polym11050777.
2. Chen, Y.; Chen, L.; Bai, H.; Li, L. Graphene oxide-chitosan composite hydrogels as broad-spectrum adsorbents for water purification. *J. Mater. Chem. A* **2013**, *1*, 1992–2001, doi:10.1039/c2ta00406b.
3. Riva, L.; Pastori, N.; Panozzo, A.; Antonelli, M.; Punta, C. Nanostructured cellulose-based sorbent materials for water decontamination from organic dyes. *Nanomaterials* **2020**, *10*, 1–18, doi:10.3390/nano10081570.
